# Active surveillance selection and 3-year durability in intermediate-risk prostate cancer following genomic testing

**DOI:** 10.1038/s41391-024-00888-y

**Published:** 2024-09-05

**Authors:** Lauren Lenz, Wyatt Clegg, Diana Iliev, Chelsea R. Kasten, Howard Korman, Todd M. Morgan, Jason Hafron, Alexander DeHaan, Carl Olsson, Ronald F. Tutrone, Timothy Richardson, Kevin Cline, Paul M. Yonover, Jeff Jasper, Todd Cohen, Robert Finch, Thomas P. Slavin, Alexander Gutin

**Affiliations:** 1 Myriad Genetics, Inc., Salt Lake City, UT USA; 2 Comprehensive Urology, Royal Oak, MI USA; 3 Wayne State University, Detroit, MI USA; 4 University of Michigan, Ann Arbor, MI USA; 5 Michigan Institute of Urology, Troy, MI USA; 6 Urologic Consultants, Wyoming, MI USA; 7 Integrated Medical Professionals, Melville, NY USA; 8 Chesapeake Urology, Towson, MD USA; 9 Wichita Urology, Pratt, KS USA; 10 Regional Urology, Shreveport, LA USA; 11 UroPartners, Chicago, IL USA

**Keywords:** Prognostic markers, Prognostic markers, Prostate cancer, Prostate cancer

## Abstract

**Background:**

Genomic testing can add risk stratification information to clinicopathological features in prostate cancer, aiding in shared medical decision-making between the clinician and patient regarding whether active surveillance (AS) or definitive treatment (DT) is most appropriate. Here we examined initial AS selection and 3-year AS durability in patients diagnosed with localized intermediate-risk prostate cancer who underwent Prolaris testing before treatment decision-making.

**Methods:**

This retrospective observational cohort study included 3208 patients from 10 study sites who underwent Prolaris testing at diagnosis from September 2015 to December 2018. Prolaris utilizes a combined clinical cell cycle risk score calculated at diagnostic biopsy to stratify patients by the Prolaris AS threshold (below threshold, patient recommended to AS or above threshold, patient recommended to DT). AS selection rates and 3-year AS durability were compared in patients recommended to AS or DT by Prolaris testing. Univariable and multivariable logistic regression models and Cox proportional hazard models were used with molecular and clinical variables as predictors of initial treatment decision and AS durability, respectively.

**Results:**

AS selection was ~2 times higher in patients recommended to AS by Prolaris testing than in those recommended to DT (*p* < 0.0001). Three-year AS durability was ~1.5 times higher in patients recommended to AS by Prolaris testing than in those recommended to DT (*p* < 0.0001). Prolaris treatment recommendation remained a statistically significant predictor of initial AS selection and AS durability after accounting for CAPRA or Gleason scores.

**Conclusions:**

Prolaris added significant information to clinical risk stratification to aid in treatment decision making. Intermediate-risk prostate cancer patients who were recommended to AS by Prolaris were more likely to initially pursue AS and were more likely to remain on AS at 3 years post-diagnosis than patients recommended to DT.

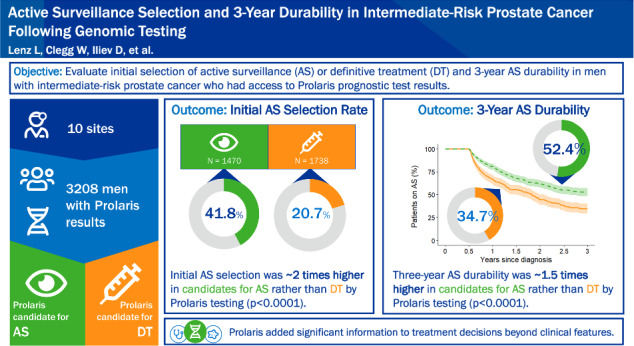

## Introduction

The National Comprehensive Cancer Network (NCCN) clinicopathological risk groups for patients with localized prostate cancer are used to guide treatment strategy, including the selection of active surveillance (AS) or definitive treatment (DT) [1]. However, these clinical risk groups are heterogeneous and may not sufficiently stratify individual risk [2]. Shared medical decision-making between the clinician and patient may be further facilitated through the use of a genomic classifier, which NCCN recommends to characterize individual personalized risk within all clinical risk groups [1].

AS is the preferred treatment option in patients with clinically defined very low–risk prostate cancer and in most patients with low-risk prostate cancer with a life expectancy ≥10 years [1–3]. High-quality evidence also supports the utility of AS in those with intermediate-risk prostate cancer with favorable outcomes based on clinicopathological features, resulting in strong recommendations from multiple societies for discussing use of AS in this group [1, 3]. In line with these recommendations, AS rates increased from ~6% in 2000 to ~40% in 2014 and close to 50% in 2019 in patients with low-risk disease [4, 5]. However, recent AS rates remain relatively low among patients with intermediate-risk, with one study reporting AS uptake in 20.4% and another in only 7.5% [5, 6].

Genomic classifiers provide an individualized disease risk estimate that may support clinician and patient confidence in AS selection. Such tests add valuable prognostic information to standard clinicopathological risk stratification measures for identifying oncologic outcomes, including adverse pathology [7–9], biochemical recurrence [10, 11], and time to switch to DT [12], and may also lead to risk reclassification, thereby resulting in better identification of AS candidates [13–17]. Incorporating genomic classifiers provides individualized, clinically actionable information that may shift risk assessment and influence AS vs DT treatment decisions in those with intermediate-risk disease [18, 19]. Multiple society guidelines support the use of tumor-based genomic classifiers and other personal risk factors to further stratify risk—especially in intermediate-risk disease— to identify patients who may be AS candidates and help inform shared decision-making [1, 2, 20].

AS reduces the risk of adverse health outcomes associated with DT, improves health-related quality of life compared to DT, and can greatly reduce the economic burden of prostate cancer [17, 21–26]. However, individuals may initially choose or switch to DT over time due to their perceived risk and uncertainty, especially among patients with intermediate-risk disease [27, 28]. PSA increases, physician recommendations, and Gleason score increases contributed to switching from AS to DT in 60% to 70% of surveyed patients [27]. Half of those surveyed reported that a personal “desire to act” drove their switch to DT [27], and it was reported that patients are likely to overestimate benefits while underestimating side effects of receiving treatment [28]. Thus, the incorporation of genomic classifiers into the shared decision-making process may help increase AS rates while reducing overtreatment-related risks and morbidity [2, 27, 28].

Here, we describe a study in patients diagnosed with localized, NCCN intermediate-risk prostate cancer who underwent Prolaris testing at diagnosis to obtain individualized risk information prior to treatment decision-making. AS selection rates and 3-year AS durability were compared in patients who were recommended to AS or DT by Prolaris, and associations between Prolaris treatment recommendation, clinical features, and initial AS selection or AS durability were analyzed.

## Methods

### Study design and patient cohort

This retrospective, observational cohort study included patients with localized NCCN intermediate-risk prostate cancer from 10 community and academic study sites (see Supplementary Materials) who underwent genomic classifier testing with Prolaris (Myriad Genetics, Inc. Salt Lake City, UT, USA) between September 2015 and December 2018. Inclusion criteria included age ≥18 years; a previous diagnosis of histologically proven, localized adenocarcinoma of the prostate; NCCN intermediate-risk status; valid Prolaris test results within 6 months of diagnosis; and ≥6 months of follow-up after diagnosis. Patients who had clinical evidence of metastasis or lymph node involvement at diagnosis, received treatment prior to diagnostic biopsy or Prolaris reporting, or used prostate cancer-specific prognostic testing other than PSA or MRI for treatment decision-making during AS were excluded (Supplementary Fig. 1). In early 2018, NCCN guidelines were updated to include favorable and unfavorable intermediate-risk categories [29]. Patients diagnosed prior to the guideline update were retrospectively assigned an NCCN favorable or unfavorable intermediate-risk status. However, as this information was not available to all patients at the time of treatment decision-making, analyses for initial AS/DT selection were not conducted based on this stratification.

During the study, consecutive, clinically tested NCCN intermediate-risk patients were identified for each site in the Prolaris clinical database at Myriad Genetics, Inc. Study sites identified eligible patients based on medical records. Clinical, pathologic, and demographic data were collected directly from the Prolaris testing and results database. Clinical data including treatment history, PSA values, imaging, and outcomes were retrospectively collected from study site patient medical records. A Waiver of Informed Consent and Full Waiver of HIPAA Authorization were obtained from Advarra IRB (Reference Number: Pro00047891). The study was performed in accordance with oversight from Advarra IRB; all participating sites were overseen for continued activities.

### Genomic classifier testing

Genomic classifier testing with Prolaris was performed at Myriad Genetics, Inc at the time of diagnosis. Prolaris uses a combined clinical cell-cycle risk (CCR) score derived from the Cancer of the Prostate Risk Assessment (CAPRA) score combined with a molecular cell-cycle progression score. The CCR score provides significant, clinically actionable prognostic information regarding metastatic risk and disease-specific mortality with validated AS and multimodal treatment thresholds [30–36]. Patients were stratified as below the AS threshold (CCR score ≤0.8, Prolaris treatment recommendation: AS) or above the AS threshold (Prolaris treatment recommendation: DT) at the time of testing.

### Statistical analysis

The Prolaris treatment recommendation was defined as whether a patient was below or above the Prolaris AS threshold (CCR score ≤0.8) and therefore recommended to AS or DT, respectively. AS selection was defined as no DT within 6 months of diagnosis; initial AS or DT status was known for all patients (AS Selection Cohort). AS selection rates for patients recommended to AS or DT by Prolaris testing were compared using a univariable conditional logistic regression model to allow for stratification by site, and are reported with 95% exact CIs. Stratified univariable and multivariable conditional logistic regression models were used to examine associations of AS selection status with Prolaris treatment recommendation, continuous CCR score, CAPRA score, and Gleason score. Univariable analyses were used to determine whether a singular variable predicted initial AS selection. Likelihood-ratio tests in the multivariable setting allowed for the evaluation of whether Prolaris added significant, independent information to clinicopathological features during initial AS/DT treatment selection. Odds ratios (ORs) with 95% profile likelihood CIs and likelihood-ratio test *p*-values are reported. ORs are reported per-unit for continuous variables.

AS durability was defined as the time between the date of diagnosis and the date of first DT. Patients without a record of DT were censored at the time of last follow up or 3-years after diagnosis, whichever occurred first. AS durability was examined among patients who were initially managed on AS and did not receive DT within 6 months of diagnosis (AS Durability Subcohort). Patients with an unknown time to first treatment were excluded from this analysis. Kaplan–Meier estimates of AS durability at 3-years post diagnosis were calculated for patients recommended to AS or DT by Prolaris testing and are reported with 95% log-log CIs. Univariable and multivariable cause-specific Cox proportional hazards models stratified by site were used to evaluate the associations of Prolaris treatment recommendation, continuous CCR score, CAPRA score, Gleason score, and subdivided NCCN intermediate risk category with AS durability. Similarly, univariable analyses were used to determine whether a singular variable is prognostic of AS durability, whereas multivariable analyses were used to evaluate whether Prolaris added significant independent information to clinicopathological features. Hazard ratios (HRs) with 95% profile likelihood CIs and likelihood-ratio test *p*-values are reported. HRs are reported per-unit for continuous variables. Time-dependent area under the receiver operator characteristic curve (AUC) values were calculated to evaluate the strength of the relationship between clinical and molecular features and time to treatment after initial AS.

Statistical analyses were performed according to a prespecified statistical analysis plan. Analyses that were not prespecified were considered exploratory. All oncologic outcomes remained blinded during these analyses. *P*-values were two-sided and considered significant at α = 0.05.

## Results

### Study cohort

This study included 3208 patients with localized NCCN intermediate-risk prostate cancer who underwent Prolaris testing within 6 months of diagnosis (AS Selection Cohort). Of those, 975 patients (30.4%) initially selected AS (Table 1). Fifteen of those who selected AS had incomplete clinical follow-up data and were excluded from AS durability analyses. Thus, 960 patients were included in the AS Durability Subcohort (Table 1). In both the AS Selection Cohort and the AS Durability Subcohort, patients who were recommended to AS by Prolaris testing tended to have more favorable clinical features than those recommended to DT (Table 1). Low (0–2), intermediate (3–5), and high (6–10) CAPRA scores were observed in 754 (51.3%), 716 (48.7%), and 0 (0%) of patients recommended AS and 112 (6.4%), 1376 (79.2%), and 250 (14.4%) of patients recommended DT by Prolaris, respectively. Patients who selected AS regardless of the Prolaris recommendation (AS Durability Subcohort) also had some clinical features that were more favorable than the entire patient cohort (AS Selection Cohort – Overall) (Table 1). A summary of the distribution of the AS Selection Cohort across the combinations of clinicopathologic variables used to classify these patients as intermediate risk by the 2018 NCCN guidelines is shown in Supplementary Table 1.

**Table 1:** Patient characteristics.

	AS Selection Cohort			AS Durability Subcohort		
	Recommended to AS^a^ (*n* = 1 470)	Recommended to DT^a^ (*n* = 1 738)	Overall (*N* = 3 208)	Recommended to AS^a^ (*n* = 605)	Recommended to DT^a^ (*n* = 355)	Full Subcohort (*N* = 960)
Age at diagnosis	66 (60–71)	68 (62–73)	67 (61–72)	67 (62–72)	69 (63–74)	68 (62–73)
Gleason Score						
3 + 3	231 (15.7%)	100 (5.8%)	331 (10.3%)	138 (22.8%)	45 (12.7%)	183 (19.1%)
3 + 4	1216 (82.7%)	1003 (57.7%)	2219 (69.2%)	462 (76.4%)	237 (66.8%)	699 (72.8%)
4 + 3	23 (1.6%)	635 (36.5%)	658 (20.5%)	5 (0.8%)	73 (20.6%)	78 (8.1%)
Percent of diagnostic biopsy cores positive for cancer	21.4% (13.1–33.3%)	35.7% (17.9–50.0%)	25.0% (16.7–41.7%)	16.7% (8.3–25.0%)	25.0% (12.9–41.7%)	16.7% (8.3–33.3%)
Diagnostic PSA (ng/ml)	5.3 (4.3–7.4)	7.2 (5.4–10.4)	6.2 (4.7–9.3)	5.8 (4.4–8.7)	7.6 (6.1–11.1)	6.5 (4.7–10.0)
Clinical T Stage						
T1	1246 (84.8%)	1447 (83.3%)	2693 (83.9%)	510 (84.3%)	315 (88.7%)	825 (85.9%)
T2	224 (15.2%)	291 (16.7%)	515 (16.1%)	95 (15.7%)	40 (11.3%)	135 (14.1%)
CAPRA score	2 (2–3)	4 (3–5)	3 (2–4)	3 (2–3)	3 (3–4)	3 (2–3)
NCCN risk category						
Favorable Intermediate	1232 (83.8%)^b^	556 (32.0%)^b^	1788 (55.7%)^b^	541 (89.4%)	169 (47.6%)	710 (74.0%)
Unfavorable Intermediate	238 (16.2%)^b^	1182 (68.0%)^b^	1420 (44.3%)^b^	64 (10.6%)	186 (52.4%)	250 (26.0%)
CCP score	−1.0 (−1.3 to −0.6)	−0.2 (-0.6 to 0.2)	-0.6 (-1.0 to -0.1)	−1.0 (−1.4 to −0.7)	−0.3 (−0.6 to 0.1)	−0.8 (−1.2 to −0.3)
CCR score	0.486 (0.210–0.657)	1.389 (1.104–1.788)	0.933 (0.495–1.446)	0.429 (0.201–0.657)	1.209 (0.999-1.512)	0.657 (0.324–1.056)
Initially managed with AS	615 (41.8%)	360 (20.7%)	975 (30.4%)	605 (100%)	355 (100%)	960 (100%)

Data are shown as *n* (%) or median (IQR).

*AS* active surveillance, *CAPRA* Cancer of the Prostate Risk Assessment, *CCP* cell-cycle progression, *CCR* cell-cycle risk; *DT* definitive treatment, *IQR* interquartile range, *PSA* prostate-specific antigen.

^a^AS and DT were recommended based on the AS threshold (CCR ≤ 0.8) provided by the Prolaris genomic classifier.

^b^NCCN guidelines were updated in 2018 to include favorable and unfavorable intermediate-risk categories. As patients underwent Prolaris testing between September 2015 and December 2018, many patients did not have had this information available at the time of diagnosis. Therefore, no analyses assessing NCCN status on initial treatment decision were performed.

### Initial AS selection

**Fig. 1:**
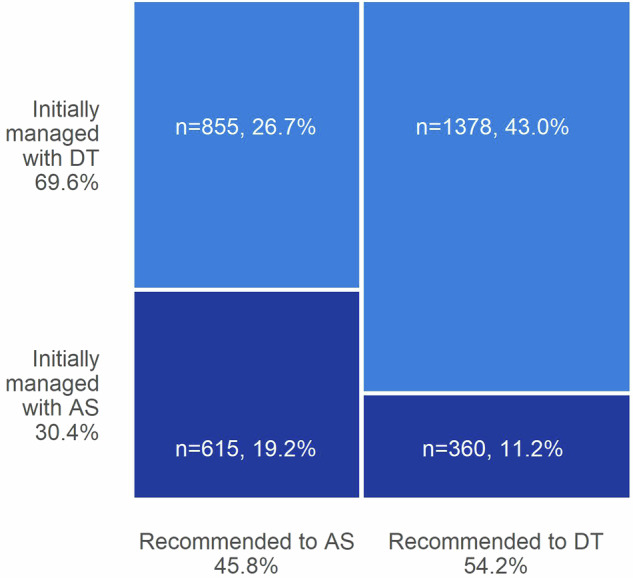
Distribution of Prolaris treatment recommendation and initial treatment decisions. The numbers and proportions of patients in the AS Selection Cohort (*N* = 3208) with a Prolaris treatment recommendation to AS (*n* = 1470) or DT (*n* = 1738) and who were initially managed with AS (dark blue) or DT (light blue) are shown in the mosaic plot. Colored areas and listed percentages represent the percent of the AS Selection Cohort. AS active surveillance, DT definitive treatment.

The AS selection rate was significantly higher for patients recommended to AS by Prolaris testing (*N* = 615/1 470, 41.8% [95% CI: 39.3–44.4%]) than for those recommended to DT (*N* = 360/1 738, 20.7% [95% CI: 18.8–22.7%]; OR 2.37 [95% CI: 2.08–2.70]; *p* < 0.0001) (Fig. 1, Table 2). A univariable logistic regression model also indicated that the continuous CCR score significantly predicted initial treatment selection (OR 0.45 [95% CI: 0.41–0.50]; *p* < 0.0001) Similarly, clinicopathological features significantly predicted initial treatment selection in exploratory univariable analyses (CAPRA: OR 0.70 [95% CI: 0.67–0.74]; *p* < 0.0001. Gleason score: 3 + 3 reference, 3 + 4 OR 0.42 [95% CI: 0.36–0.48], 4 + 3 OR 0.13 [95% CI 0.10–0.16]; *p* < 0.0001) (Table 2).

**Table 2:** Prolaris treatment recommendation and continuous CCR score add significant information to AS/DT selection decision after accounting for clinicopathological variables.

Predictor variable	OR (95% CI)	*p*-value
Univariable models		
Prolaris treatment recommendation^a^		<0.0001
AS	2.37 (2.08–2.70)	
DT	Reference	
Continuous CCR score^b^	0.45 (0.41–0.50)	<0.0001
CAPRA^b^	0.70 (0.67–0.74)	<0.0001
Gleason score		<0.0001
3+3	Reference	
3+4	0.42 (0.36–0.48)	
4+3	0.13 (0.10–0.16)	
**Bivariable Prolaris treatment recommendation + CAPRA**		
Prolaris treatment recommendation^a^		<0.0001
AS	1.71 (1.50–1.95)	
DT	Reference	
CAPRA^b^	0.81 (0.77–0.86)	<0.0001
**Bivariable Prolaris treatment recommendation + Gleason score**		
Prolaris treatment recommendation^a^		<0.0001
AS	1.71 (1.50–1.95)	
DT	Reference	
Gleason score		<0.0001
3+3	Reference	
3+4	0.45 (0.39–0.52)	
4+3	0.18 (0.14–00.23)	
Bivariable continuous CCR + CAPRA		
Continuous CCR score^b^	0.47 (0.43–0.53)	<0.0001
CAPRA^b^	0.97 (0.92–1.03)	0.52
Bivariable continuous CCR + Gleason score		
Continuous CCR score^b^	0.57 (0.51–0.63)	<0.0001
Gleason score		<0.0001
3+3	Reference	
3+4	0.47 (0.40–0.54)	
4+3	0.25 (0.20–0.32)	

Univariable and multivariable conditional logistic regression models including molecular and clinical variables were generated to predict initial binary AS/DT treatment status, stratified by site, in the AS Selection Cohort.

*AS* active surveillance, *CAPRA* Cancer of the Prostate Risk Assessment, *CCR* cell-cycle risk, *CI* confidence interval, *DT* definitive treatment, *OR* odds ratio.

^a^Prolaris treatment recommendations to AS or DT were based on the AS threshold (CCR score ≤0.8) provided by the Prolaris genomic classifier.

^b^OR is reported per-unit for continuous variables.

Exploratory multivariable logistic regression analyses confirmed that Prolaris treatment recommendation remained significant after accounting for CAPRA score (OR 1.71 [95% CI: 1.50–1.95], *p* < 0.0001) or Gleason score (OR 1.71 [95% CI: 1.50–1.95], *p* < 0.0001). Similarly, continuous CCR score remained significant after accounting for CAPRA score (OR 0.47 [95% CI: 0.43–0.53], *p* < 0.0001) or Gleason score (exploratory analysis; OR 0.57 [95% CI: 0.51–0.63], *p* < 0.0001) (Table 2).

### Three-year AS durability

**Fig. 2:**
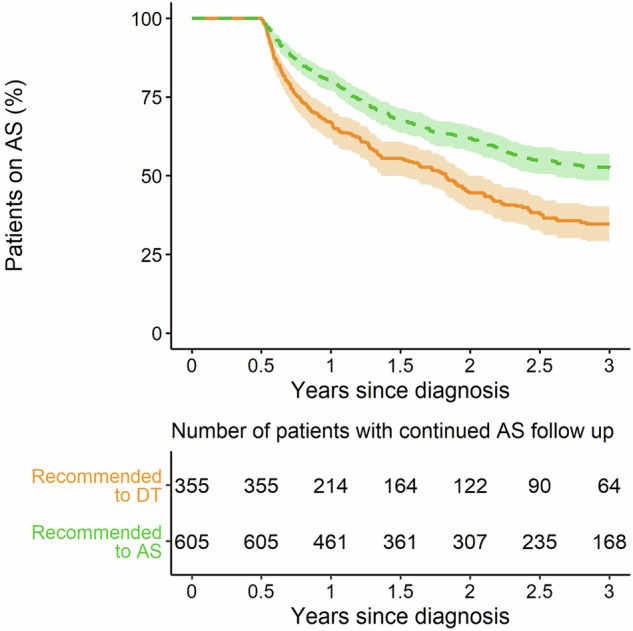
AS durability by Prolaris treatment recommendation over 3 years among patients in the AS Durability Subcohort. Kaplan–Meier estimates of AS durability over 3 years after diagnosis were calculated for patients who initially selected AS (*N* = 960) who were recommended to AS (dashed green; *n* = 605) or recommended to DT (solid orange; *n* = 355) by Prolaris testing. Shaded areas represent 95% CI. The number of patients at risk at each time point is shown below the graph. AS active surveillance, DT definitive treatment.

The median follow-up time observed in patients with no recorded DT during uncensored follow up was 2.8 years (IQR 1.8–3.80). The 3-year AS durability was significantly higher among patients recommended to AS by Prolaris testing; patients recommended to AS by Prolaris testing were ~1.5 times more likely to remain on AS 3 years after diagnosis (3-year AS durability in patients recommended AS: 52.4% [95% CI, 48.0–56.7%]; recommended DT: 34.7% [95% CI, 29.2–40.3%]; HR, 0.62 [95% CI, 0.51–10.74], *p* < 0.0001) (Fig. 2, Table 3). A univariable Cox proportional hazards model also indicated that AS durability was significantly predicted by continuous CCR score (HR 1.50 [95% CI: 1.27–1.77], *p* < 0.0001). Similarly, clinicopathological features and NCCN intermediate risk stratification based on clinicopathological features significantly predicted AS durability in exploratory univariable analyses (CAPRA: HR 1.16 [95% CI: 1.06–1.26]; *p* < 0.01. Gleason score: 3 + 3 reference, 3 + 4 HR 1.40 [95% CI, 1.13–1.74], 4 + 3 HR 1.88 [95% CI 1.36–2.53]; *p* < 0.01. NCCN risk category: favorable intermediate reference, unfavorable intermediate HR 1.59 [95% CI: 1.30–1.94], *p* < 0.0001) (Table 3). Time-dependent AUCs are summarized in Supplementary Table 2.

**Table 3:** Prolaris treatment recommendation and continuous CCR score add significant information to AS durability after accounting for clinicopathological variables.

Predictor variable	HR (95% CI)	*p*-value
Univariable models		
Prolaris treatment recommendation^a^		<0.0001
AS	0.62 (0.51–0.74)	
DT	Reference	
Continuous CCR score^b^	1.50 (1.27–1.77)	<0.0001
CAPRA^b^	1.16 (1.06–1.26)	0.002
Gleason score		0.004
3+3	Reference	
3+4	1.40 (1.13–1.74)	
4+3	1.88 (1.36–2.53)	
NCCN risk category		<0.0001
Favorable Intermediate	Reference	
Unfavorable Intermediate	1.59 (1.30–1.94)	
Bivariable Prolaris treatment recommendation + CAPRA		
Prolaris treatment recommendation^a^		<0.001
AS	0.63 (0.52–0.77)	
DT	Reference	
CAPRA^b^	1.03 (0.94–1.12)	0.65
Bivariable Prolaris treatment recommendation + Gleason score		
Prolaris treatment recommendation^a^		<0.0001
AS	0.64 (0.53–0.77)	
DT	Reference	
Gleason score		0.10
3+3	Reference	
3+4	1.32 (1.07–1.65)	
4+3	1.39 (1.00–1.87)	
Bivariable Prolaris treatment recommendation + NCCN Status		
Prolaris treatment recommendation^a^		0.0007
AS	0.69 (0.57–0.84)	
DT	Reference	
NCCN risk category		0.01
Favorable Intermediate	Reference	
Unfavorable Intermediate	1.33 (1.08–1.62)	
Bivariable continuous CCR + CAPRA		
Continuous CCR score^b^	1.61 (1.37–1.90)	<0.001
CAPRA^b^	0.95 (0.87–1.04)	0.47
Bivariable continuous CCR + Gleason score		
Continuous CCR score^b^	1.50 (1.27–1.76)	<0.0001
Gleason score		0.10
3+3	Reference	
3+4	1.33 (1.07–1.66)	
4+3	1.20 (0.87–1.62)	
Bivariable continuous CCR + NCCN Status		
Continuous CCR Score^b^	1.33 (1.13–1.56)	0.005
NCCN Status		0.03
Favorable Intermediate	Reference	
Unfavorable Intermediate	1.31 (1.07–1.60)	

Univariable and multivariable Cox proportional hazards models with molecular and clinical variables were generated to predict time to first treatment, stratified by site, in the AS Durability Subcohort.

*AS* active surveillance, *CAPRA* Cancer of the Prostate Risk Assessment, *CCR* cell-cycle risk, *CI* confidence interval, *DT* definitive treatment, *OR* odds ratio.

^a^Prolaris treatment recommendations to AS or DT were based on the AS threshold (CCR score ≤0.8) provided by the Prolaris genomic classifier.

^b^OR is reported per-unit for continuous variables.

Exploratory multivariable Cox proportional hazard analyses confirmed that Prolaris treatment recommendation remained significant after accounting for CAPRA score (HR 0.63 [95% CI, 0.52–0.77]; *p* < 0.001), Gleason score (HR 0.64 [95% CI, 0.53–0.77]; *p* < 0.0001) or NCCN risk category (HR 0.69 [95% CI, 1.08–1.62]; *p* = 0.0007) (Table 3). Similarly, continuous CCR score remained significant after accounting for CAPRA score (HR 1.61 [95% CI, 1.37–1.90]; *p* < 0.001), Gleason score (exploratory analysis; HR 1.50 [95% CI, 1.27–1.76]; *p* < 0.0001) or NCCN risk category (exploratory analysis; HR 1.33 [95% CI, 1.13–1.56]; *p* = 0.005) (Table 3). Although neither CAPRA nor Gleason score add significant prognostic information to either Prolaris treatment recommendation or continuous CCR score, NCCN risk category does add some independent information to Prolaris treatment recommendation (HR 1.33 [95% CI, 1.08–1.62]; *p* = 0.01) and continuous CCR score (HR 1.31 [95% CI 1.07–1.60]; *p* = 0.03) (Table 3). Time-dependent AUCs for these bivariable models are summarized in Supplementary Table 2.

## Discussion

This study evaluated AS selection rates and 3-year AS durability among patients with NCCN intermediate-risk prostate cancer who underwent Prolaris testing at diagnosis to obtain individualized risk information prior to treatment decision-making. The findings from this study suggest that the genomic classifier provided by Prolaris helps to increase AS selection rates in those for whom AS is appropriate by providing individualized risk information, especially in patients with intermediate risk. In this study, those with intermediate-risk prostate cancer and access to Prolaris testing results had high rates of AS selection. Notably, patients recommended to AS by Prolaris testing initially chose AS at roughly twice the rate of those recommended to DT (41.8% vs 20.7%). These rates exceed those previously observed over the same time period (2015–2018) in patients with intermediate risk (7.5–10.4%) [5, 6]. Additionally, patients with intermediate risk recommended to AS by Prolaris testing chose AS at twice the rate of patients with intermediate risk in the AUA Quality (AQUA) registry in 2019 (41.8% vs 20.4%) [5], even though the current study largely predated guideline changes noting AS utility in patients with favorable intermediate risk [1, 29]. These findings are in line with a previous prospective study reporting that AS rates increased with genomic classifier testing in individuals with newly diagnosed localized prostate cancer [19].

The current results suggest that additional risk information from Prolaris testing helps to increase AS uptake and durability. Although clinicopathological features are beneficial in initial AS selection and durability, Prolaris added significant independent information to these measures. Notably, Gleason and CAPRA score did not add significant information to Prolaris, but NCCN favorable vs. unfavorable risk category added significant information to Prolaris for AS durability. This may be due to Prolaris deriving clinical information from CAPRA, which is inclusive of Gleason score [37], whereas NCCN risk groups were adapted from D’Amico risk grouping [1, 38]. Although the information captured by CAPRA and D’Amico overlaps, the two risk methods do not completely correlate and may provide discrepant identification of intermediate- and high-risk patients [37].

Large cohort studies performed in patients with predominately very low– and low-risk prostate cancer have reported AS durability rates of 43% to 81% across 2 to 15 years of follow-up, with the switch to DT increasing over the length of the follow-up period [39–41]. Risk classification has been shown to play a role in determining the endurance of AS selection, with higher rates of switching to DT among individuals with intermediate risk (40.8% to >78.5%) than those with very low– or low-risk disease (29.8–44.9%) over 3 to 10 years of follow-up [42–44]. In one study, patients with higher-risk disease based on genomic classifier testing were twice as likely to switch to DT as their counterparts with lower genomic risk, but the study was underpowered to detect significant effects of genomic information [44].

In this study, AS durability at 3 years was 52.4% in patients recommended to AS by Prolaris testing and 34.7% in those recommended to DT. These early durability rates are in line with previously published rates, and the higher rate of AS durability among patients recommended to AS by Prolaris testing highlights the utility of genomic classifiers in treatment decision-making. Additionally, multivariable analyses showed that the Prolaris treatment recommendation added significant information to clinicopathological features, such as Gleason, CAPRA scores, and NCCN risk category. These findings are in accordance with previous studies showing the utility of genomic classifiers in informing a switch to DT in subsets of assessed cohorts [12, 45]. These results suggest that incorporating genomic classifiers into shared decision-making processes provides valuable personalized information for weighing risks against benefits when determining initial treatment and whether to escalate treatment.

Strengths of this study include the relatively large data set, with > 3000 samples, which were relatively evenly distributed by Prolaris treatment recommendation to AS or DT. Samples were collected from 10 community and academic study sites, adding to the generalizability of the findings. The study adds to the knowledge on initial AS selection rates and the rate of exiting AS for DT in patients with intermediate-risk prostate cancer. The use of multivariable analyses in this study help to reinforce the utility of genomic risk stratification when considering initial AS selection and switching from AS to DT. This study also adds to the general literature supporting the utility of Prolaris, including prognostic value for metastasis, disease specific mortality, and treatment benefit [31–35], as well as the molecular cell cycle progression component having a meaningful effect on physician treatment recommendations [46, 47].

Limitations of this study include the retrospective observational nature of the study and patient selection bias. Additionally, this study was performed relatively soon after the Prolaris AS threshold was developed and when AS was largely underutilized, particularly in patients with NCCN intermediate-risk prostate cancer [48]. Much of the data in this study was collected prior to the stratification of NCCN favorable and unfavorable intermediate-risk groups. Therefore, we did not assess whether the favorable/unfavorable classification provided significant information during initial patient treatment choices. Additionally, race and ethnicity data that were collected in this study were optional and self-reported, and nearly all patients (96.8%) did not provide these data. Previous studies in low-risk populations reported that Black patients are more likely to switch to DT than White patients at 2- and 10-years post diagnosis [12, 49]; this study was not able to examine whether Black patients with intermediate risk are more likely to switch to DT sooner than White patients with intermediate risk. Future studies should examine the relationships among the AS and DT recommendations from the binary Prolaris AS threshold, continuous CCR score, and NCCN favorable/unfavorable intermediate-risk stratification and the effects of race and ethnicity on AS selection and durability in patients with intermediate-risk prostate cancer. Another limitation is that this study was not able to include additional outcomes, such as survey data that would indicate how Prolaris was incorporated into treatment decision making or oncologic outcomes that demonstrate long-term safety of choosing AS. These outcomes may be considered in future studies.

In conclusion, the results of this study show that intermediate-risk prostate cancer patients who were recommended to AS by Prolaris are more likely to initially pursue AS and are more likely to remain on AS at 3 years post-diagnosis than patients recommended to DT. These results reinforce the utility of genomic classifiers such as Prolaris for providing individualized information that can aid in risk stratification and inform shared medical decision-making, particularly in those with intermediate-risk disease.

## Supplementary information

Supplemental Material

## Data Availability

The datasets used and/or analyzed in this study are available from the corresponding author on reasonable request.
