# Supplementary material for: Active surveillance selection and 3-year durability in intermediate-risk prostate cancer following genomic testing
Source: Prostate Cancer Prostatic Dis. 2024 Sep 5;28(2):427–34. doi: 10.1038/s41391-024-00888-y (PMC12106071; doi:10.1038/s41391-024-00888-y)
Supplement: Supplementary file 1 — Supplemental Material [file 41391_2024_888_MOESM1_ESM.docx]

**Active Surveillance Selection and 3-Year Durability in Intermediate-Risk Prostate Cancer Following Genomic Testing**

Lauren Lenz, Wyatt Clegg, Diana Iliev, Chelsea R. Kasten, Howard Korman, Todd M. Morgan, Jason Hafron, Alexander DeHaan, Carl Olsson, Ronald F. Tutrone Jr., Timothy Richardson, Kevin Cline, Paul M. Yonover, Jeff Jasper, Todd Cohen, Robert Finch, Thomas P. Slavin Jr., Alexander Gutin

**Supplementary Materials**

**Participating sites.**

Arkansas Urology

Chesapeake Urology

Comprehensive Urology

Integrated Medical Professionals

Michigan Institute of Urology

Regional Urology

University of Michigan

Urologic Consultants

URO Partners

Wichita Urology

**Supplementary Figure 1.** Patient flow diagram.


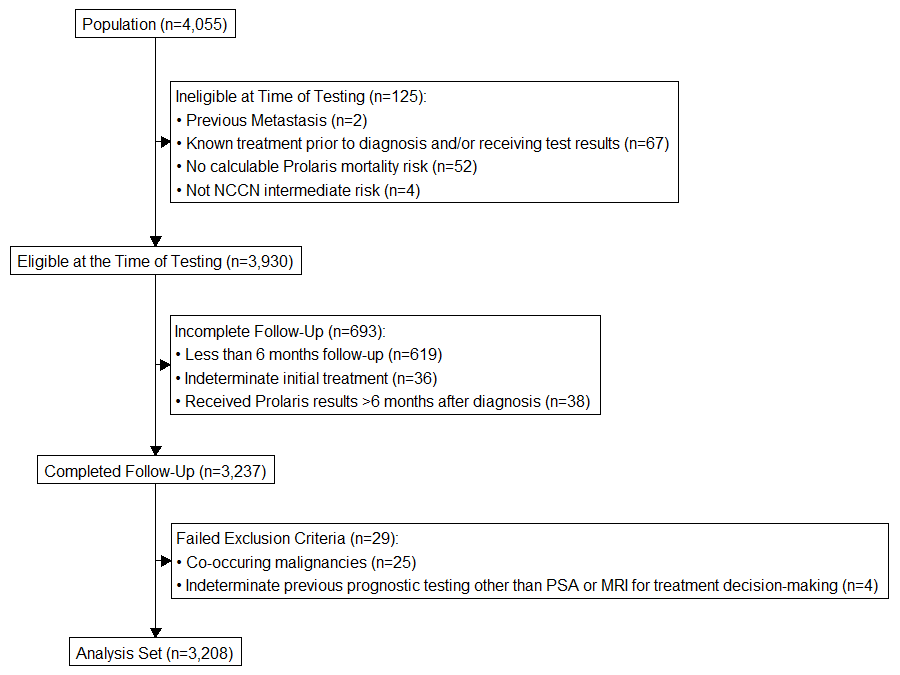


**Supplementary Table 1.** Distribution of the AS Selection Cohort (n = 3 208) across the combinations of clinicopathologic variables used to classify patients as having favorable intermediate or unfavorable intermediate disease by the 2018 NCCN guidelines.

| **NCCN** | **Gleason Score** | **T Stage** | **% Positive Cores** | **PSA** | **N (%)** |
| --- | --- | --- | --- | --- | --- |
| Favorable Intermediate | 3+3 | T1-T2a | <50% | 10-20 | 241 (7.5%) |
| Unfavorable Intermediate | 3+3 | T1-T2a | >50% | 10-20 | 16 (0.5%) |
| Favorable Intermediate | 3+3 | T2b-T2c | <50% | <10 | 52 (1.6%) |
| Unfavorable Intermediate | 3+3 | T2b-T2c | >50% | <10 | 12 (0.4%) |
| Unfavorable Intermediate | 3+3 | T2b-T2c | Any | 10-20 | 10 (0.3%) |
| Favorable Intermediate | 3+4 | T1-T2a | <50% | <10 | 1495 (46.6%) |
| Unfavorable Intermediate | 3+4 | T1-T2a | >50% | <10 | 364 (11.3%) |
| Unfavorable Intermediate | 3+4 | T1-T2a | Any | 10-20 | 266 (8.3%) |
| Unfavorable Intermediate | 3+4 | T2b-T2c | Any | <10 | 78 (2.4%) |
| Unfavorable Intermediate | 3+4 | T2b-T2c | Any | 10-20 | 16 (0.5%) |
| Unfavorable Intermediate | 4+3 | ≤T2c | Any | ≤20 | 658 (20.5%) |

**Supplementary Table 2.** Time-dependent AUC calculated at three years post-diagnosis from models predicting time to treatment in patients originally managed with active surveillance.

| Model | AUC |
| --- | --- |
| Univariate |  |
| Prolaris Treatment Recommendation | 58.0% |
| CCR Score | 58.0% |
| CAPRA | 54.1% |
| Gleason Score | 53.4% |
| NCCN Risk Category | 54.0% |
| Bivariate |  |
| Prolaris Treatment Recommendation + CAPRA | 57.9% |
| Prolaris Treatment Recommendation + Gleason Score | 59.3% |
| Prolaris Treatment Recommendation + NCCN Risk Category | 58.6% |
| CCR Score + CAPRA | 58.2% |
| CCR Score + Gleason Score | 58.4% |
| CCR Score + NCCN Risk Category | 57.9% |
